# Characterization of antifungal C‐type lectin receptor expression on murine epithelial and endothelial cells in mucosal tissues

**DOI:** 10.1002/eji.202149192

**Published:** 2021-06-23

**Authors:** Mark H.T. Stappers, Christina Nikolakopoulou, Darin L. Wiesner, Raif Yuecel, Bruce S. Klein, Janet A. Willment, Gordon D. Brown

**Affiliations:** ^1^ Aberdeen Fungal Group Institute of Medical Sciences University of Aberdeen Aberdeen UK; ^2^ Department of Biosciences Medical Research Council Centre for Medical Mycology at the University of Exeter Exeter UK; ^3^ Department of Pediatrics School of Medicine and Public Health University of Wisconsin‐Madison Madison WI USA; ^4^ Iain Fraser Cytometry Centre Institute of Medical Sciences University of Aberdeen Aberdeen UK; ^5^ Exeter Centre for Cytomics (EXCC) Department of Biosciences College of Life and Environmental Sciences University of Exeter Exeter EX4 4QD UK

**Keywords:** C‐type lectin, Epithelial cells, Endothelial cells, Immunity, Innate

## Abstract

Our data reveal that selection of enzymes for generating single cell suspensions from murine tissues influences detection of surface expression of antifungal CLRs. Using a method that most preserves receptor expression, we show that non‐myeloid expression of antifungal CLRs is limited to MelLec on endothelial cells in murine mucosal tissues.

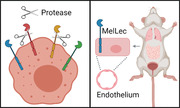

C‐type lectin receptors (CLRs) constitute a family of pattern recognition receptors (PRRs), many of which are involved in immunity [1]. Of particular interest are signaling CLRs whose encoding genes are located in the Dectin‐1 and Dectin‐2 cluster of receptors [2, 3]. Many of these CLRs play key roles in immunity to fungi, including *Candida*, *Aspergillus*, and *Cryptococcus* [1, 2, 3]. These receptors, Dectin‐1, Dectin‐2, Mincle, MCL, and MelLec, recognize ligands in the fungal cell wall and are able to induce intracellular signaling directly through integral, or membrane associated, signaling domains or by association with a signaling partner [1].

Most of these “antifungal” CLRs are thought to be expressed by myeloid cells, but there is some evidence suggesting that these receptors are also expressed on nonmyeloid cells including endothelial and epithelial cells [4, 5, 6, 7, 8]. Indeed, we recently identified MelLec as a novel antifungal CLR that was primarily expressed by endothelial cells [9]. Epithelial and endothelial cells are known to be involved in controlling inflammatory, immune and regenerative responses in several diseases (including asthma) but our understanding of the role of these cells during fungal infections is still poorly understood. We therefore undertook a systematic exploration of antifungal CLR expression on epithelial and endothelial cells at mucosal surfaces of the respiratory, gastrointestinal, and genital‐urinary tract, which are commonly exposed to fungal pathogens.

In the literature, a range of proteolytic enzymes have been used for the generation of single cell suspensions of tissues. However, these treatments can affect the levels of surface molecules and have functional consequences [10]. Little is known about the impact of such treatment on the expression and function of CLRs. We therefore investigated the effect of commonly used proteolytic enzymes on the ability to detect surface expression of CLRs. For these evaluation experiments, we made use of NIH3T3 cells overexpressing Dectin‐1, Mincle, MCL, MelLec, or Dectin‐2, which were treated with a variety of the most commonly used tissue‐dissociation enzymes. Following enzymatic treatment, we assessed CLR expression by flow cytometry and observed that detection of Dectin‐1 expression was markedly decreased upon incubation with most enzymes (Fig. 1A and Supporting Information Fig. S1A and B). The reduction in Dectin‐1 surface expression was not unexpected, as its protease‐sensitivity has been characterized previously [1, 11]. MCL was similarly decreased upon incubation with most enzymes (Fig. 1B), but detection of both MCL and Dectin‐1 was least affected following treatment using the lung dissociation kit (Fig. 1A and B and Supporting Information Fig. S1B and C). Detection of Mincle was markedly reduced upon incubation with all enzymes tested (Fig. 1C and Supporting Information Fig. S1D). In contrast, detection of MelLec and Dectin‐2 was largely unaffected by digestion with any of the enzymes tested (Fig. 1D and E and Supporting Information Fig. S1E and F). Thus, enzymatic digestion can significantly influence the ability to detect expression of some CLRs on cells.

**Figure 1:**
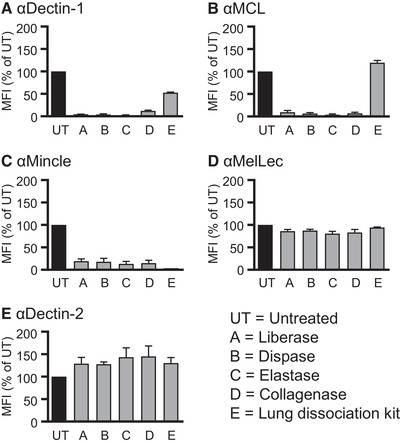
Effect of enzymatic digestion on C‐type lectin receptor expression on NIH3T3 cells. Median fluorescence intensity (MFI) of CLR expression, as determined by flow cytometry, on NIH3T3 cells overexpressing Dectin‐1 (A), MCL (B), Mincle (C), MelLec (D), and Dectin‐2 (E) after incubation with enzymes (as described) represented as percentage of untreated (UT) cells. Error bars represent mean ± SEM of pooled data from three independent experiments.

Using the lung dissociation kit, which had the least effect on the ability to detect CLR surface expression, we next assessed the expression of these receptors on murine lung epithelial and endothelial cells ex vivo. As we have previously observed [9], expression of MelLec could be detected on naïve lung CD45^–^CD31^+^ endothelial cells (Fig. 2A and Supporting Information Fig. S2A). However, we did not detect MelLec expression on CD45^–^CD31^–^EpCAM^+^ epithelial cells (Fig. 2B). To confirm this result, we investigated MelLec expression on the three major epithelial cell subtypes in the lung. Epithelial cell subset specific promoters driving EGFP expression were used to identify type II alveolar (*Sftpc*), club (*Scgb1a1*), and ciliated (*Foxj1*) epithelial cells. These data confirm that expression of MelLec is absent on all three major epithelial populations (Supporting Information Fig. S2B and C).

**Figure 2:**
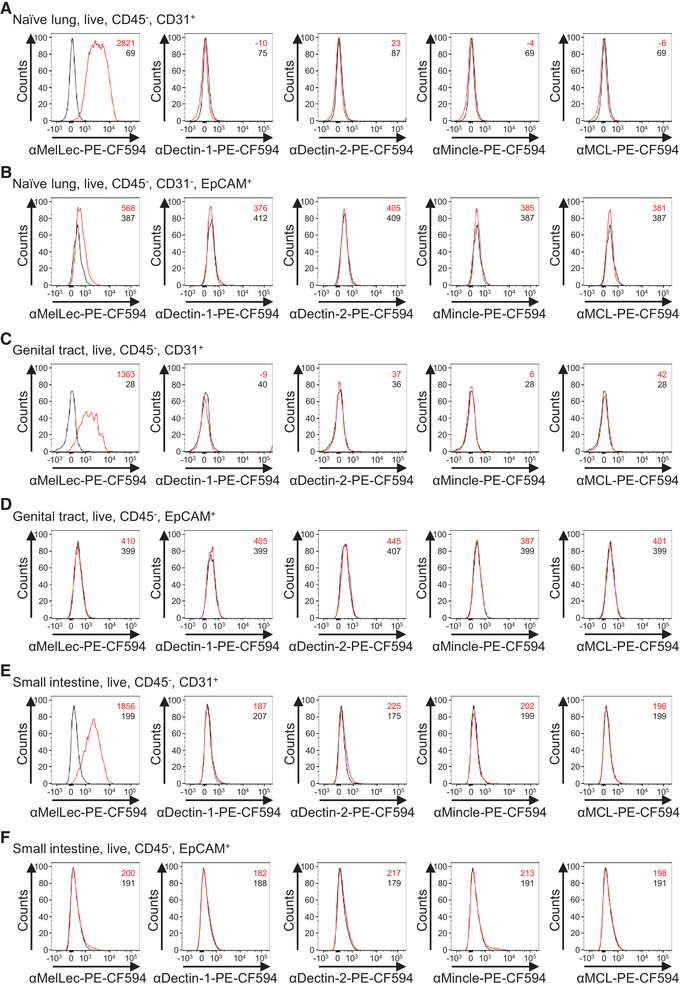
Expression of C‐type lectin receptors on epithelial and endothelial populations of the murine mucosal surfaces. Flow‐cytometric plots of CLR expression (representative of two independent experiments, first experiment three individual mice, second experiment on cells pooled from two mice) on single, live, CD45^–^CD31^+^ endothelial cells (A) and CD45^−^EpCAM^+^ epithelial cells (B) in the naïve lung. Flow‐cytometric plots of CLR expression (cells pooled from five mice, one experiment. CLEC‐1 expression, three independent experiments, second and third experiment on cells pooled from three mice) on single, live, CD45^–^CD31^+^ endothelial cells (C) and CD45^−^EpCAM^+^ epithelial cells (D) in the genital‐urinary tract. Flow‐cytometric plots of CLR expression (cells pooled from three mice, one experiment. CLEC‐1 expression, three independent experiments, second and third experiment on cells pooled from two mice) on single, live, CD45^–^CD31^+^ endothelial cells (E) and CD45^−^EpCAM^+^ epithelial cells (F) in the small intestine. Lines represent expression of CLR (red) and isotype control (black).

To determine if expression of MelLec could be modulated under inflammatory conditions, we intratracheally infected mice with *Aspergillus fumigatus* and assessed receptor expression after 24 h. We determined that expression of MelLec was unaltered on lung CD45^–^CD31^+^ endothelial cells (Supporting Information Fig. S2D), and remained undetectable on CD45^–^EpCAM^+^ cells (Supporting Information Fig. S2E). Thus, expression of MelLec is unaltered by inflammatory conditions.

Several reports have suggested that other anti‐fungal CLRs can be expressed by endothelial and/or epithelial cells [4, 5, 6, 7, 8]. We therefore explored the expression of Dectin‐1, Dectin‐2, Mincle, and MCL in naïve and infected mouse lungs, as described above. Notably, we did not detect expression of any of these receptors on endothelial or epithelial under any conditions tested (Fig. 2A and B and Supporting Information Fig. S2D and E). We cannot exclude, however, that expression of CLRs may be upregulated under other inflammatory conditions. Although the inability to detect Mincle expression could be due to enzymatic digestion (Fig. 1C), the absence of MCL supports our conclusion, as these receptors form a heterodimer that is required for surface expression [1], that is, absence of MCL would correlate with absence of Mincle. Thus, we conclude that MelLec is the only antifungal CLR we examined that is expressed on nonmyeloid cells in the lung in the mouse.

Finally, we explored CLR expression on nonmyeloid cells at two other mucosal tissues frequently in contact with fungi, the genital‐urinary and gastrointestinal tract. Similar to the lung, only MelLec was detected on naïve CD45^–^CD31^+^ endothelial cells, and no CLRs were detected on CD45^–^EpCAM^+^ epithelial cells of the genital‐urinary tract (Fig. 2C and D and Supporting Information Fig. S2F). Unfortunately, the lung dissociation kit was not suitable for generating viable, single cell populations of the small intestine for analysis and we had to resort to using Collagenase VIII to analyze this tissue. As before, only MelLec was detected on CD45^–^CD31^+^ endothelial cells, and no CLRs were detected on CD45^–^EpCAM^+^ epithelial cells isolated from the gastrointestinal tract (Fig. 2E and F and Supporting Information Fig. S2G). The caveat with the latter experiment being the significantly reduced ability to detect Dectin‐1, MCL, and Mincle using this tissue digestion method (Fig. 1 and Supporting Information Fig. S1). Thus, the expression of these antifungal CLRs on mucosal nonmyeloid cells appears limited to MelLec on endothelial cells. Previous reports have also suggested expression of Dectin‐1 on a wider range of nonmyeloid cells in humans [4, 5, 6, 7], raising the possibility of differential expression of this CLR between species. This is a focus of our future studies.

In summary, our data reveal that the selection of enzymes for generating single cell suspensions from murine tissues influences the detection of surface expression of antifungal CLRs, which may have consequences in subsequent in vitro analyses [1, 11]. Using a digestion method that most preserves receptor surface expression, we show that nonmyeloid expression of antifungal CLRs is limited to MelLec on endothelial cells in mucosal tissues of the respiratory, gastrointestinal, and genital‐urinary tracts in mice, at least at the protein level by flow cytometry.

## Conflict of interest

The authors declare no commercial or financial conflict of interest.

### Peer review

The peer review history for this article is available at https://publons.com/publon/10.1002/eji.202149192

## Supporting information

Supporting Information

## Data Availability

The data that support the findings of this study are available from the corresponding author upon reasonable request.
