# Supplementary material for: Characterization of antifungal C‐type lectin receptor expression on murine epithelial and endothelial cells in mucosal tissues
Source: Eur J Immunol. 2021 Jun 23;51(9):2341–4. doi: 10.1002/eji.202149192 (PMC8593890; doi:10.1002/eji.202149192)
Supplement: Supplementary file 1 — Supporting Information [file EJI-51-2341-s001.pdf]

Supporting information

Materials and methods

Animals

Wildtype, female C57BL/6 mice (8-12 weeks old) were bred in-house under specific pathogen-free conditions at the University of Aberdeen. CreERT2 mice (Scgb1a1-CreER^{TM}, Sftpc-CreER^{T2}) crossed to CRE-inducible GFP mouse (Rosa26-LSL-Cas9 knock-in on B6J) and Foxj1Cre$^{ERT2::GFP}$ mice were bred in-house under specific pathogen-free conditions at the University of Wisconsin-Madison and treated with intraperitoneal injections of 2 mg tamoxifen for four consecutive days to induce GFP expression in epithelial populations, type II alveolar (*Sftpc*), club (*Scgb1a1*) and ciliated cells (*Foxj1*). All animal experiments conformed to the animal care and welfare protocols approved by UK Home Office (project license P79B6F297) or the Institutional Animal Care and Use Committee in compliance with the relevant ethical guidelines.

Cell lines and enzymatic treatment

NIH3T3 fibroblasts expressing murine MelLec (CLEC1A), Dectin-1 or combinations of FcRγ and mDectin-2, mMCL and/or mMincle were generated by retroviral transduction as described previously [1, 2]. NIH3T3 cells were cultured in DMEM medium supplemented with 10% heat-inactivated foetal calf serum, 100 units/mL penicillin, 0.1 mg/mL streptomycin, and 2 mM L-glutamine, at 37°C and 5% CO_2.

NIH3T3 cells were lifted using lidocaine (8mg/mL) / EDTA (5mM) solution and incubated with enzymes Liberase TM (25 µg/mL, Roche), Elastase (0.7 mg/mL, Worthington), Dispase I (0.08 mg/mL, Sigma-Aldrich), Collagenase VIII (1 mg/mL, Sigma-Aldrich) and enzymes A and D (Lung dissociation kit, Miltenyi Biotec), for 30 minutes at 37°C, followed by staining and acquisition by flow cytometry (see below).

Strains, growth conditions, and murine infection model

A. fumigatus isolate 13073 (American Type Culture Collection) was grown on potato dextrose agar for 7 days at 37°C. Conidia were harvested with PBS containing 0.1% Tween-80 and passed through a 40-µm nylon filter, and washed in PBS before use. Conidial counts were obtained using a haemocytometer. To assess receptor

expression during pulmonary infection, $1*10^7$ *A. fumigatus* ATCC 13073 conidia in 40 µL were administered to the caudal oropharynx of anesthetized mice. Mice were culled 24 hours after infection and lungs were isolated for further processing.

Isolation of single cells from mucosal tissues

The lungs, small intestine and genital-urinary tract of mice were dissected and cut into small pieces. Single cell suspensions were obtained by incubation of lung and genital tract tissues with enzymes A and D according to manufacturer's protocol (Lung dissociation kit, Miltenyi Biotec) in RPMI (Gibco). The small intestine was incubated with 1 mg/mL Collagenase VIII (Sigma-Aldrich). After 30 minutes of incubation at 37°C, whilst shaking, the cells were dissociated using the gentleMACS™ Dissociator (Miltenyi Biotec). Subsequently, the cells were strained through a 70 µm nylon cell strainer (Fisher Scientific). Red blood lysis was performed using PharmLyse (BD Biosciences), and the remaining isolated cells were used in accordance with experimental requirements. Lungs of CreERT2 mice were incubated with 50% Dispase (Corning) / 50% Collagenase I (Worthington) and single cells suspensions were prepared as above.

Flow cytometric analysis

Cells from the various tissues or NIH3T3 fibroblasts were incubated in staining buffer (PBS with 0.5% (w/v) BSA and 5 mM EDTA) containing anti-CD16/CD32 (10 µg/mL, Clone 2.4G2, prepared in house) at 4°C, prior to the addition of anti-CLR antibodies (5 µg/mL, prepared in house unless otherwise indicated): anti-MelLec-Biotin (clone 14c8), anti-Dectin-1-Biotin (clone 2a11), anti-Dectin-2-Biotin (clone 11e4), anti-Mincle-Biotin (clone 4a9, a kind gift from Sho Yamasaki [3]), anti-MCL-Biotin (clone 3a4), or isotype controls. Tissue samples were also stained with anti-CD45.2-FITC (Clone 104, BD Biosciences), anti-CD326-APC (EpCAM; Clone G8.8, eBioscience), anti-CD31-PE-Cyanine7 (PECAM-1; Clone 390, eBioscience). After washing away unbound antibody, cells were then incubated with Streptavidin-PE-CF594 (BD Biosciences), in staining buffer at 4ºC. Incubation with fixable viability dye eFluor-780 (eBioscience) in PBS at 4ºC was used to assess cell viability. Adequate flow cytometry controls such as unstained, isotype and Fluorescence Minus One (FMO) were prepared to identify the boundary and specificity of the signal. Finally, cells were fixed using formaldehyde

(1%, v/v) and acquired by flow cytometry (LSRII and LSR Fortessa, Becton Dickinson). Guidelines for the use of flow cytometry in immunological studies were followed [4].

Data analysis

Data were analysed and presented using FlowJo v10 (Becton Dickinson). Graphical representation of the data was presented using GraphPad Prism.

Supporting information references

1 **Stappers, M. H. T. et al,** *Nature* 2018. **555**: 382-386.
2 **Kerscher, B. et al,** *Eur J Immunol* 2016. **46**: 381-389.
3 **Miyake, Y. et al,** *Immunity* 2013. **38**: 1050-1062.
4 **Cossarizza, A. et al,** *Eur J Immunol* 2019. **49**: 1457-1973.

Supporting information figure 1

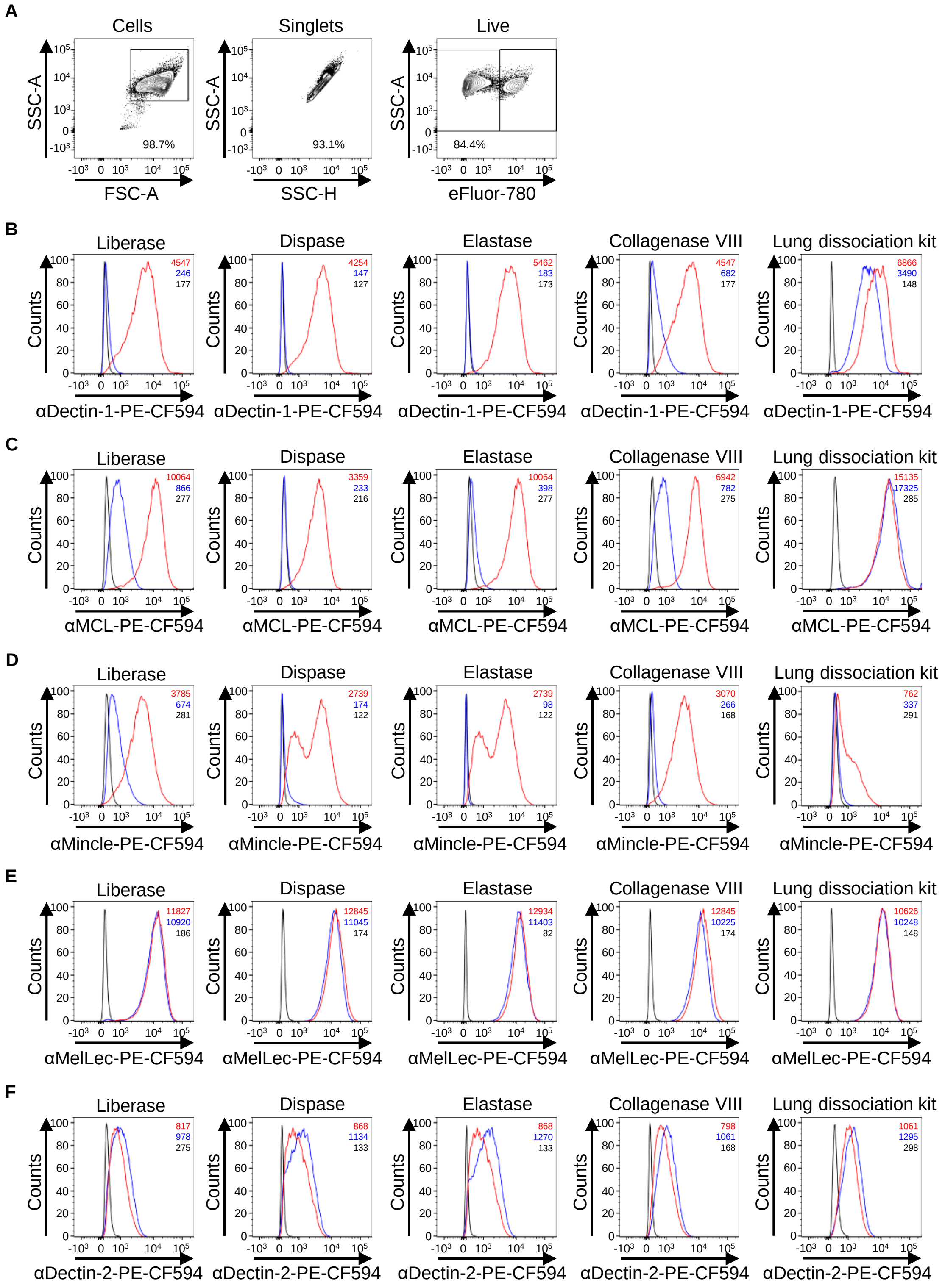

Supporting information figure 1. Effect of enzymatic digestion on CLR expression on NIH3T3 cells. A, Gating strategy for NIH3T3 cells, selecting for single and live NIH3T3 cells. Representative histograms of CLR expression on NIH3T3 CLR overexpressing cells after incubation with enzymes (as described, representative of 3 independent experiments), Dectin-1 (B), MCL (C), Mincle (D), MelLec (E) and Dectin-2 (F). Lines represent NIH3T3 CLR overexpressing cells untreated (red), after enzymatic treatment (blue) and untreated NIH3T3 parental cells without CLR of interest (black).

Supporting information figure 2

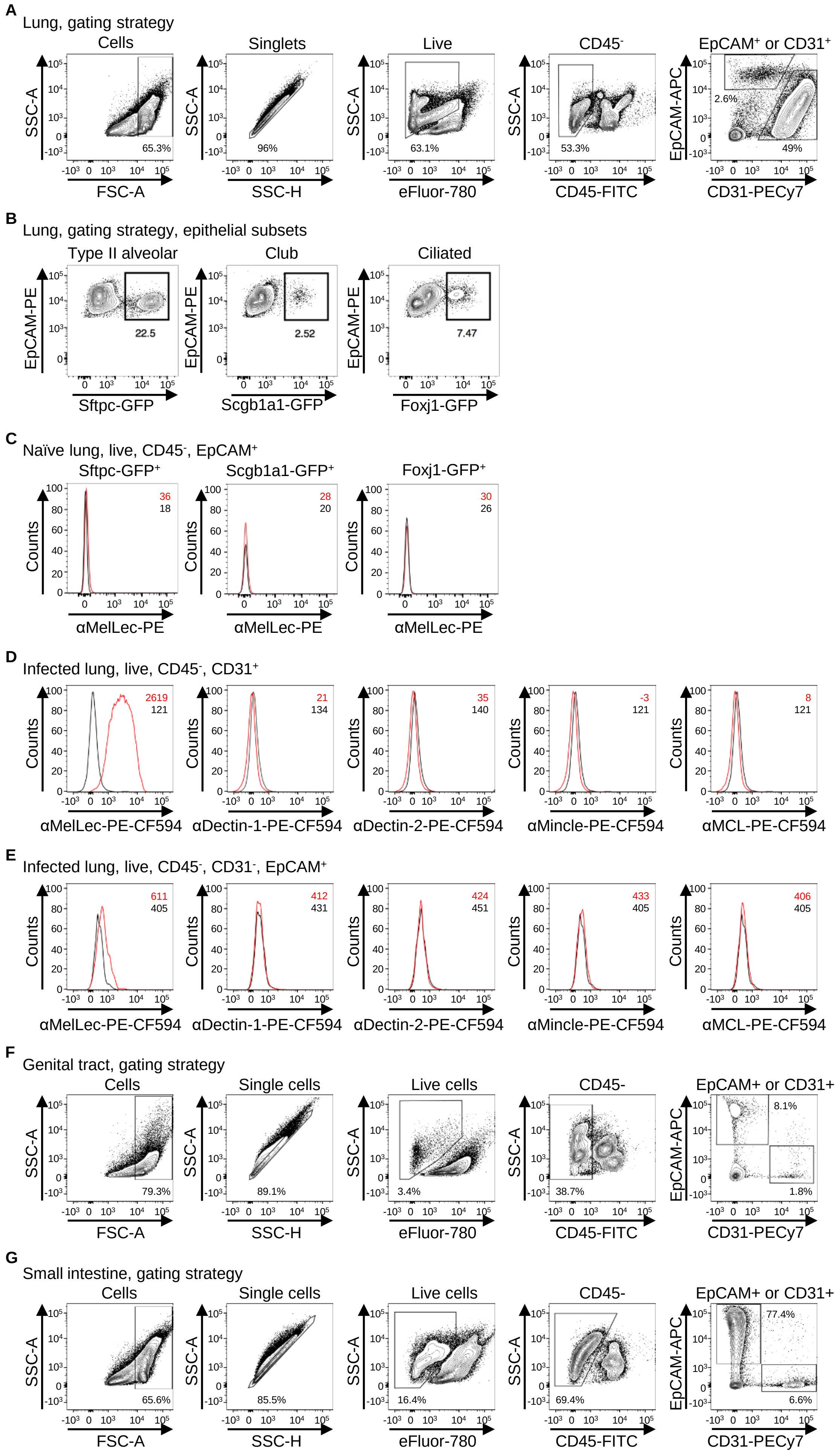

Supporting information figure 2. Gating strategy for the identification of cells from the murine mucosal surfaces. A, Gating strategy for the identification of single, live, $CD45^-$, $CD31^+$ endothelial cells or $EpCAM^+$ epithelial cells from digested lung tissue (representative of 3 independent experiments, n = 3 mice). B, Gating strategy for the identification of single, live, $CD45^-$, $EpCAM^+$ type II alveolar (Sftpc-GFP^+), club (Scgb1a1-GFP^+), ciliated (Foxj1-GFP^+) epithelial cells from digested lung tissue (representative of 1 experiment, n = 3 mice). C, Representative flow-cytometric plots of MelLec expression on single, live, $CD45^-EpCAM^+$ type II alveolar (Sftpc-GFP^+), club (Scgb1a1-GFP^+), ciliated (Foxj1-GFP^+) epithelial cells in the naïve lung (3 individual mice, 1 experiment). Representative flow-cytometric plots of CLR expression (as described, 3 individual mice, 1 experiment) under inflammatory conditions, on single, live, $CD45^-CD31^+$ endothelial cells (D) and $CD45^-EpCAM^+$ epithelial cells (E) in the *A. fumigatus* infected lung. Lines represent expression of CLR (red) and isotype control (black) or FMO control (black, c). F, Gating strategy for the identification of single, live, $CD45^-$, $EpCAM^+$ epithelial cells or $CD31^+$ endothelial cells from digested genital-urinary tissue (on cells pooled from 5 mice, 1 experiment). G, Gating strategy for the identification of single, live, $CD45^-$, $EpCAM^+$ epithelial cells or $CD31^+$ endothelial cells from digested small intestine tissue (on cells pooled from 3 mice, 1 experiment).
